# RXFP1 Receptor Activation by Relaxin-2 Induces Vascular Relaxation in Mice *via* a Gα_i2_-Protein/PI3Kß/γ/Nitric Oxide-Coupled Pathway

**DOI:** 10.3389/fphys.2018.01234

**Published:** 2018-09-04

**Authors:** Xiaoming Lian, Sandra Beer-Hammer, Gabriele M. König, Evi Kostenis, Bernd Nürnberg, Maik Gollasch

**Affiliations:** ^1^Experimental and Clinical Research Center (ECRC), Charité – University Medicine Berlin and Max Delbrück Center for Molecular Medicine in the Helmholtz Association, Berlin, Germany; ^2^Department of Pharmacology and Experimental Therapy, Institute of Experimental and Clinical Pharmacology and Toxicology, Eberhard Karls University Hospitals and Clinics, and Interfaculty Center of Pharmacogenomics and Drug Research (ICePhA), Tübingen, Germany; ^3^Institute for Pharmaceutical Biology, University of Bonn, Bonn, Germany; ^4^Medical Clinic for Nephrology and Internal Intensive Care, Charité Campus Virchow Klinikum, Berlin, Germany

**Keywords:** serelaxin, relaxin-2, endothelial Gα_i2_, NO, RXFP1 receptor, perivascular-adipose tissue, ADRF

## Abstract

**Background:** Relaxins are small peptide hormones, which are novel candidate molecules that play important roles in cardiometablic syndrome. Relaxins are structurally related to the insulin hormone superfamily, which provide vasodilatory effects by activation of G-protein-coupled relaxin receptors (RXFPs) and stimulation of endogenous nitric oxide (NO) generation. Recently, relaxin could be demonstrated to activate G_i_ proteins and phosphoinositide 3-kinase (PI3K) pathways in cultured endothelial cells *in vitro*. However, the contribution of the G_i_-PI3K pathway and their individual components in relaxin-dependent relaxation of intact arteries remains elusive.

**Methods:** We used Gα_i2_- (*Gnai2*^-/-^) and Gα_i3_-deficient (*Gnai3*^-/-^) mice, pharmacological tools and wire myography to study G-protein-coupled signaling pathways involved in relaxation of mouse isolated mesenteric arteries by relaxins. Human relaxin-1, relaxin-2, and relaxin-3 were tested.

**Results:** Relaxin-2 (∼50% relaxation at 10^-11^ M) was the most potent vasodilatory relaxin in mouse mesenteric arteries, compared to relaxin-1 and relaxin-3. The vasodilatory effects of relaxin-2 were inhibited by removal of the endothelium or treatment of the vessels with *N* (G)-nitro-L-arginine methyl ester (L-NAME, endothelial nitric oxide synthase (eNOS) inhibitor) or simazine (RXFP1 inhibitor). The vasodilatory effects of relaxin-2 were absent in arteries of mice treated with pertussis toxin (PTX). They were also absent in arteries isolated from *Gnai2*^-/-^ mice, but not from *Gnai3*^-/-^ mice. The effects were not affected by FR900359 (Gα_q_ protein inhibitor) or PI-103 (PI3Kα inhibitor), but inhibited by TGX-221 (PI3Kβ inhibitor) or AS-252424 (PI3Kγ inhibitor). Simazine did not influence the anti-contractile effect of perivascular adipose tissue.

**Conclusion:** Our data indicate that relaxin-2 produces endothelium- and NO-dependent relaxation of mouse mesenteric arteries by activation of RXFP1 coupled to G_i2_-PI3K-eNOS pathway. Targeting vasodilatory G_i_-protein-coupled RXFP1 pathways may provide promising opportunities for drug discovery in endothelial dysfunction and cardiometabolic disease.

## Introduction

Cardiometabolic syndrome is a combination of metabolic dysfunctions mainly characterized by insulin resistance, impaired glucose tolerance, dyslipidemia, hypertension, and central adiposity. Relaxins were initially viewed as pregnancy hormones because originally identified to be produced by the ovary corpus luteum in pregnant women (Bell et al., 1987). Later relaxins were also found to be produced in the heart, in the mammary gland, in the endometrium (Kakouris et al., 1992; Du et al., 2010), in the placental trophoblast cells and in the prostate (Hansell et al., 1991). Relaxins are structurally related to the insulin hormone superfamily, consisting of two peptide chains derived from a common processor that are linked by two disulfide bridges. They act through different relaxin family peptide receptors (RXFPs) and signaling pathways (Dessauer and Nguyen, 2005). Humans express three forms of relaxins encoded by three separate genes, that is, relaxin-1 (*RLX1*), relaxin-2 (*RLX2*), and relaxin-3 (*RLX3*) genes. Relaxin-1 and relaxin-2 are the major circulating relaxin isoforms in humans and other mammals (Grossman and Frishman, 2010), while relaxin-3 is expressed at high levels in the nucleus incertus in human and rodent brains, where it has been postulated to act locally as a neuropeptide (Goto et al., 2001).

In recent years, there has been increasing interest in the possible role of relaxins in cardiometabolic syndrome. Relaxin treatment reduces food intake in rats (McGowan et al., 2010), reverses insulin resistance and restored endothelial-dependent vasodilatation in high-fat-diet mice (Bonner et al., 2013). Moreover, RXFPs have been suggested to represent potential targets for anti-anxiety and anti-obesity drugs (Halls et al., 2007). Meanwhile, serelaxin (RLX030), the drug represents the recombinant form of human relaxin-2, also shows promising effects in the therapeutic process in patients with hypertension (Papadopoulos et al., 2013), acute heart failure (AHF) (Teerlink et al., 2013), and ischemic heart disease (Parikh et al., 2013), most likely through its vasodilatory, antifibrotic, and antigenic properties (Conrad, 2010).

Relaxins are capable of inducing vasodilation in human (Fisher et al., 2002) and rodent microvessels (Conrad, 2010). The effects are mediated *via* specific G-protein-coupled receptors (GPCRs), the relaxin family peptide receptor (RXFP) 1–4 (Bathgate et al., 2006). RXFP1 is widely expressed in heart, kidney, lung, liver, blood vessels, and various areas of the brain. This GPCR is considered to be the fundamental RXFP receptor to mediate relaxin effects in the cardiovascular system by complex mechanisms and intracellular signaling pathways (Bani-Sacchi et al., 1995; Nistri and Bani, 2003). Cell culture experiments indicate that relaxin could activate endothelial nitric oxide synthase (eNOS) *via* a pertussis toxin (PTX)-sensitive G_i_-PI3K-dependent pathway (Halls et al., 2006; Novak et al., 2006; van der Westhuizen et al., 2008). However, it is unknown whether this pathway is relevant to vasodilation of intact vessels. It is also unknown which G proteins are involved in this pathway. RXFP2 activates adenylate cyclase in recombinant systems, but physiological responses are sensitive to pertussis toxin. RXFP3 and RXFP4 resemble more conventional peptide ligand receptors and both inhibit adenylate cyclase, and in addition RXFP3 activates Erk1/2 signaling *in vitro* (Bathgate et al., 2006). Although G_i_-proteins have been suggested to play important roles in cardiovascular disease, in particular in ischemia reperfusion injury (Eisen et al., 2004), the involvements of specific G_i_ isoform(s) [Gα_i1_, Gα_i2_, and/or Gα_i3_] and vasodilatory GPCRs are unknown. This is particularly important since there are only few reports that GPCRs are capable to utilize G_i_-coupled signaling pathways to cause vasodilation (i.e., for bradykinin, beta_2_ adrenergic agonists, thrombin) (Liao and Homcy, 1993; Ciccarelli et al., 2007; Vanhoutte et al., 2017). It is therefore not surprising that current research is focused on the identification of novel compounds and GPCRs which can utilize G_i_-signaling pathways to produce potent relaxations. Since relaxins are endogenous hormones, which could exhibit vascular effects *via* G_i_ protein-coupled pathways (Halls et al., 2006; Novak et al., 2006; van der Westhuizen et al., 2008), our study was aimed to examine the putative vasodilatory effect of relaxins and the involved G- and PI3K-dependent signaling pathways. In this study, we compared the sensitivity of the three human relaxins 1–3 in eliciting relaxation of mouse mesenteric arteries and tested the hypothesis that the NO-dependent vasodilatory effect of relaxins is mediated by the activation of endothelial RXFP1 receptors, which are coupled to vasodilatory G_i2_-PI3K-eNOS signaling pathways. Finally, we also tested whether the RXFP1 pathway is involved in the periadventitial control of arterial tone by perivascular adipose tissue (PVAT).

## Materials and Methods

### Mice

Experiments were conducted according to the National Institutes of Health Guide for the Care and Use of Laboratory Animals, and the protocols were previously approved by the local Animal Care and Use Committee from Berlin LAGeSo (G0132/14). Animal experiments used 10- to 14-week-old mice of either sex and were housed in groups of four to six animals in cages with nesting material, mouse lodges, and open access to water and feed, at 23°C with a 12 h/12 h circadian cycle. Most experiments were performed using male wild-type (WT, C57BL/6N) mice. To define Gα_i_ isoforms involved in relaxin effects, we used female Gα_i2_-deficient (*Gnai2*^-/-^) and Gα_i3_-deficient (*Gnai3*^-/-^) mice and respective littermate (+/+) controls. The generation and basal phenotypic characterization of Gα_i2_-deficient and Gα_i3_-deficient mice are described elsewhere (Rudolph et al., 1995; Gohla et al., 2007; Ezan et al., 2013; Wiege et al., 2013).

### Preparation of PTX-Treated Animals

Male wild-type C57BL/6 mice (20–25 g, 8–12 weeks) were maintained according to national guidelines for animal care at the animal facility. Mice were injected intraperitoneally with 150 μg/kg body weight pertussis toxin (PTX) or NaCl solution (0.9 %) as vehicle control 48 h before use (Köhler et al., 2014).

### Measurement of Vascular Reactivity by Wire Myography

The second branches of mesenteric arteries were isolated from mice under inhalation anesthesia with isoflurane and killed by cervical dislocation. The vessels were then quickly transferred to cold (4°C) and oxygenated (95% O_2_/5% CO_2_) physiological salt solution (PSS) containing (in mmol/L): 119 NaCl, 4.7 KCl, 1.2 KH_2_PO_4_, 25 NaHCO_3_, 1.2 MgSO_4_, 11.1 glucose, 1.6 CaCl_2_, and then dissected into 2 mm rings whereby perivascular fat and connective tissue were either intact [(+) PVAT or removed (-) PVAT] without damaging the adventitia. Each ring was positioned between two stainless steel wires (diameter 0.0394 mm) in a 5-mL organ bath of a Small Vessel Myograph (DMT 610M, Danish Myo Technology, Aarhus, Denmark) (Fésüs et al., 2007). The software Chart5 (AD Instruments, Ltd., Spechbach, Germany) was used for data acquisition and display. The rings were pre-contracted and equilibrated for 30 min until a stable resting tension has been acquired. In some vessels, the endothelium was removed mechanically by a whisker or an air bubble (Fésüs et al., 2007). Endothelium integrity or functional removal was confirmed by the presence or absence, respectively, of the relaxant response to 1 μM acetylcholine (ACh) on phenylephrine (PE 1 μM) pre-contracted arteries. Following PSS wash, the pharmacological drugs were applied. After a waiting period of 30 min, PE and subsequently relaxin-1 or -2 or -3 or vehicle (PSS) was added to the bath solution. Relaxations induced by relaxins were expressed as percentage relaxations obtained with ACh (100%) or as percentage relaxations of PE contractions. Contractions induced by PE were expressed as percentage tension obtained with KCl-PSS (100%) containing (in mmol/L): 63.7 NaCl, 60 KCl, 1.2 KH_2_PO_4_, 25 NaHCO_3,_ 1.2 Mg_2_SO_4_, 11.1 glucose, and 1.6 CaCl_2_. During the experiments, relaxin was applied for at least 5 min, and data were recorded to ensure that relaxins achieved their maximal effects (Willcox et al., 2013). All drugs were added to the bath solution (PSS).

### Materials

FR900359 was isolated as described previously (Schrage et al., 2015). Salts and drugs have been purchased from Sigma-Aldrich (Germany) with the exception of PTX and TGX 221, which were obtained from Merck Millipore (Calbiochem) (Germany) and PI-103 from Enzo Life Sciences. The PI3K inhibitors TGX-221, AS-252424, and PI-103 were administered at submaximal concentrations to achieve biological effects (Ali et al., 2008; Tsvetkov et al., 2016a). All drugs were freshly dissolved on the day of the experiment according to the material sheet. The relaxin peptides have been purchased from Sigma-Aldrich (Germany). Relaxins were dissolved in water. The following concentrations were used: relaxin-1 at 10 pM or at 100 pM, relaxin-2 at 10 pM or at 100 pM or from 1 pM to 10 nM, relaxin-3 at 10 pM or at 100 pM.

### Statistical Analyses

Results are presented as mean ± SEM. Data were analyzed statistically using the GraphPad Prism 7 software (GraphPad Software, San Diego, CA, United States). Unpaired Student’s *t*-tests or ANOVA were used where appropriate. A value of *P* less than 0.05 was considered statistically significant; *n* represents the number of arteries tested.

## Results

### Relaxation of Mesenteric Arteries by Relaxin-1, Relaxin-2, and Relaxin-3

**FIGURE 1:**
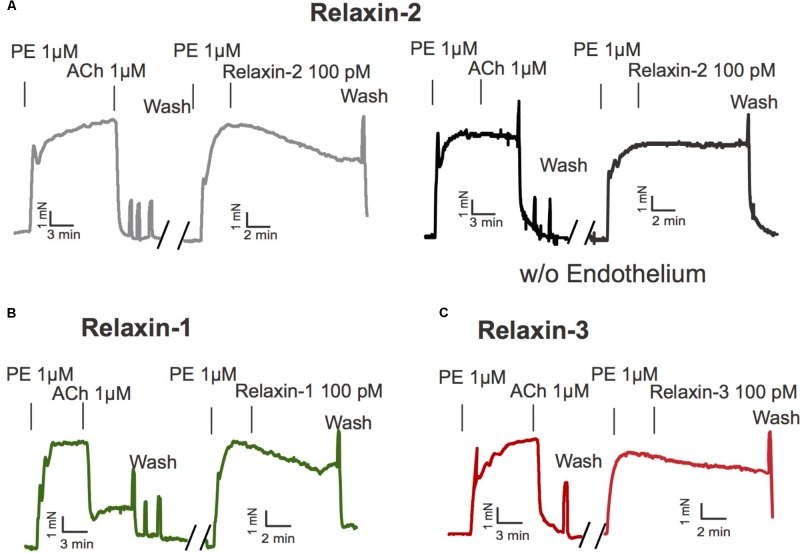
Effects of relaxins on mesenteric arteries. **(A)** Original representative recording for relaxin-2 induced relaxation with (left) and without endothelium (right). **(B)** Original representative recording for relaxin-1 induced relaxation. **(C)** Original representative recording for relaxin-3 induced relaxation. PE, phenylephrine. ACh, acetylcholine. For numbers of experiments, see **Figure 2**.

**FIGURE 2:**
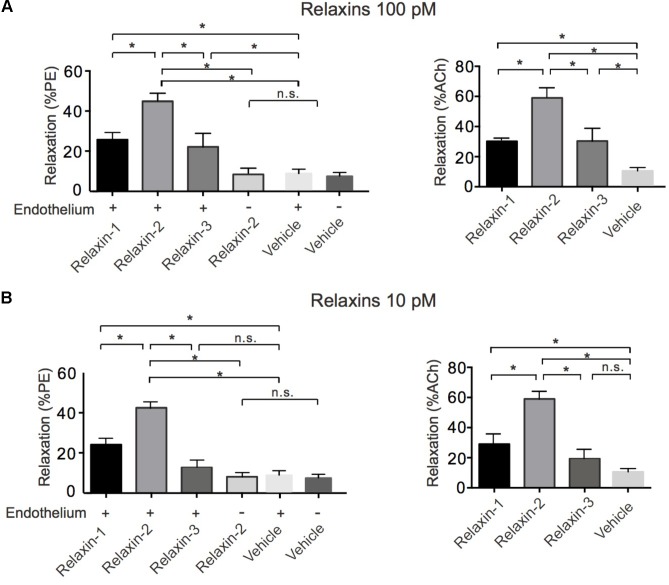
Summary data for relaxations induced by relaxins. **(A)** Summary data for % relaxation (PE) by 100 pM relaxin-1 (*n* = 6 rings out of 5 mice), 100 pM relaxin-2 (*n* = 8 out of six mice), 100 pM relaxin-3 (*n* = 6 out of five mice), vehicle control in vessels with endothelium (*n* = 6 out of four mice), and vehicle control in vessels without endothelium (*n* = 6 out of four mice) and for % relaxation relative to ACh (100%) response. Relaxin-1 (*n* = 6 out of five mice), relaxin-2 (*n* = 8 out of six mice), relaxin-3 (*n* = 6 out of five mice), and vehicle control in vessels with endothelium (*n* = 6 out of four mice). **(B)** Summary data for % relaxation (PE) by 10 pM relaxin-1 (*n* = 6 rings out of four mice), 10 pM relaxin-2 (*n* = 7 out of four mice), 10 pM relaxin-3 (*n* = 6 out of four mice), vehicle control in vessels with endothelium (*n* = 6 out of four mice), and vehicle control in vessels without endothelium (*n* = 6 out of four mice) and for % relaxation relative to ACh (100%) response. Relaxin-1 (*n* = 6 out of four mice), relaxin-2 (*n* = 7 out of four mice), relaxin-3 (*n* = 6 out of four mice), and vehicle control in vessels with endothelium (*n* = 6 out of four mice). ^∗^*P* < 0.05 using one-way ANOVA followed by Bonferroni multiple comparisons test; n.s., not significant.

We first evaluated the vasoactive properties of the three different relaxins, that is, relaxin-1, relaxin-2, and relaxin-3. Isolated mesenteric artery rings were pre-contracted by phenylephrine (PE 1 μM) and exposed to acetylcholine (ACh 1 μM) for control. After wash-out of these substances, the vessels were re-exposed to PE and subsequently incubated with relaxin-1, relaxin-2, or relaxin-3 (at 10^-10^ M each) in separate vessels (Willcox et al., 2013) (**Figure 1**). All three relaxins produced relaxations (**Figure 2A**). However, relaxin-2, at the same concentration tested, was more effective than relaxin-1 or relaxin-3 in producing vasodilatory effects (**Figure 2A**). Importantly, this increased efficiency for relaxin-2 was even more pronounced at 10-fold lower concentrations (10^-11^ M; **Figure 2B**). Compared to ACh, relaxations in response to relaxin-1, relaxin-2, and relaxin-3 were slow and delayed (**Figure 1**). In order to exclude the possibility that relaxations by relaxins result from a spontaneous loss of the pre-contraction level, we performed control experiments with vehicle (PSS) in arteries with and without endothelium. Our results argue against this possibility (**Figure 2**). Removal of the endothelium abolished both ACh and relaxin-2 relaxations (**Figure 1A**, right). These data indicate that relaxins are powerful peptide hormones to produce relaxations, probably utilizing an endothelium-dependent mechanism distinct from ACh. Since relaxin-2 was the strongest vasodilator, we continued the following mechanistic studies using this relaxin peptide.

### Relaxin-2 Causes RXFP-Induced Relaxation via eNOS/NO Signaling Without Involvement of Gα_q_ Proteins

**FIGURE 3:**
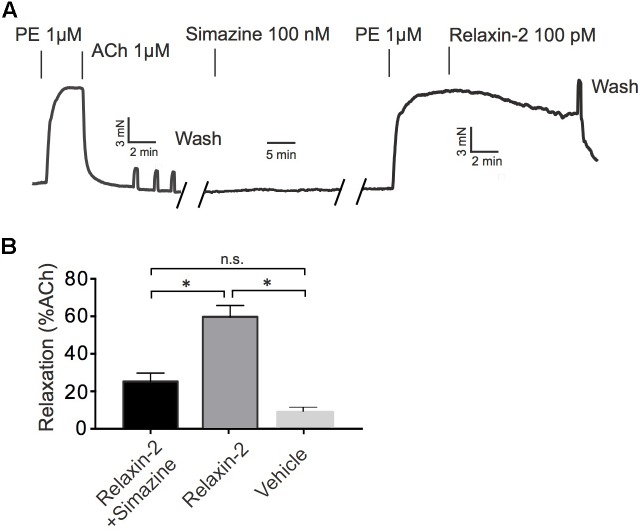
Effects of simazine on relaxin-2 induced relaxations. **(A)** Original representative recording on relaxin-2 induced relaxation in the presence of 100 nM simazine. **(B)** Summary data for relaxin-2 induced relaxation in the presence of 100 nM simazine (*n* = 9 out of six mice). ^∗^*P* < 0.05 using one-way ANOVA followed by Bonferroni multiple comparisons test; n.s., not significant.

**FIGURE 4:**
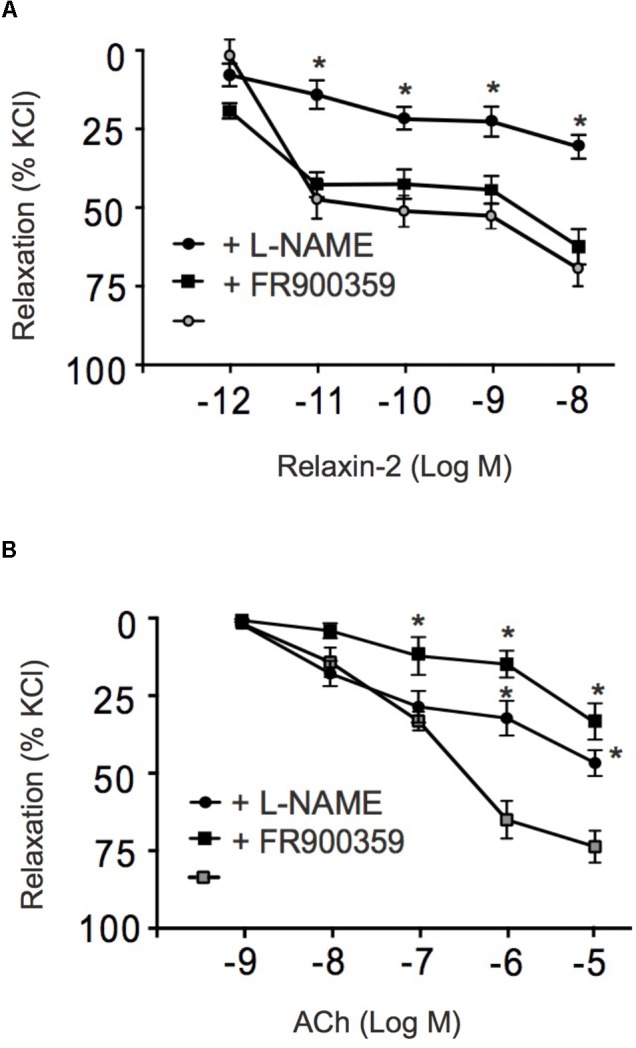
Effects of FR900359 and L-NAME on acetylcholine and relaxin-2 induced relaxations. **(A)** Summary data for relaxin-2 induced relaxationsin arteries in the presence of L-NAME (100 μM, 30 min) (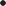, *n* = 6 out of four mice), in the presence of FR900359 (100 nM, 30 min) (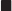, *n* = 6 out of four mice), and in non-treated arteries (control group) (∙, *n* = 6 out of four mice). **(B)** Summary data for ACh-induced relaxations in arteries in the presence of L-NAME (100 μM) (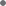, *n* = 6 out of four mice), in the presence of FR900359 (100 nM) (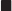, *n* = 6 out of four mice), and non-treated arteries (control group) (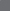, *n* = 6 out of four mice). ^∗^*P* < 0.05 for relaxin-2 + L-NAME vs. control or ACh + L-NAME vs. non-treated vessels or ACh + FR900359 vs. non-treated vessels; repeated-measures two-way ANOVA, followed by Bonferroni *post hoc* test.

**Figure 3** shows that the RXFP1 blocker simazine (100 nM) inhibited relaxations induced by relaxin-2. **Figure 4** shows that the Gα_q_ protein inhibitor FR900359 (100 nM) (Schrage et al., 2015) inhibited concentration-dependent relaxations by ACh (**Figure 4B**), but had no effect on relaxin-2-dependent relaxations (**Figure 4A**). In these experiments, pre-tension was induced by KCl-PSS, which causes membrane depolarization to cause Ca^2+^ influx into vascular smooth muscle cells and hence vasocontraction independently of Gα_q_ protein activation (Wirth et al., 2008). In contrast, the eNOS inhibitor L-NAME (100 μM) inhibited both relaxin-2- and ACh-induced relaxations (**Figures 4A,B**, respectively). Together, the data indicate that both relaxin-2 and ACh produce relaxation by an endothelial-dependent mechanism involving eNOS/NO release. However, whereas ACh utilizes an eNOS/NO signaling pathway involving muscarinic receptors coupled to G_q_ proteins (Kruse et al., 2012), relaxin-2 stimulates RXFP1 receptors coupled to G proteins other than G_q_ to produce eNOS/NO-dependent relaxation.

### Involvement of G_i_ Proteins in Relaxin-2-Induced Relaxation

**FIGURE 5:**
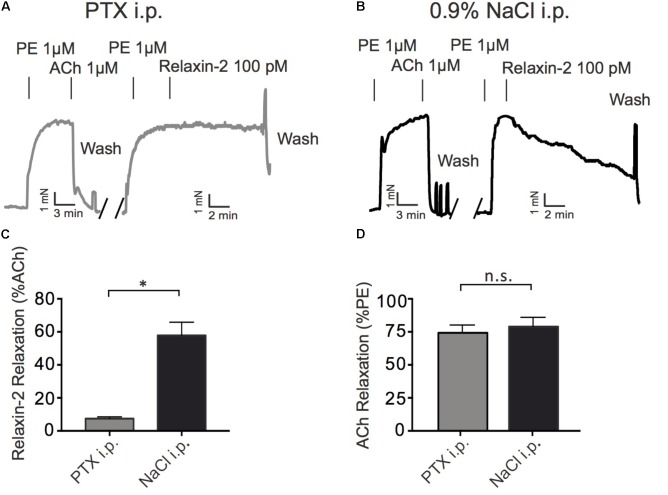
Effects of treatment of mice with pertussis toxin (PTX) or sham (0.9% NaCl) on relaxin-2 relaxations. **(A)** Original representative recording of relaxin-2 induced relaxations in arteries of PTX treated mice. **(B)** Original representative recording of relaxin-2 induced relaxations in arteries of 0.9% NaCl treated (control) mice. **(C)** Summary data of relaxin-2 induced relaxation. **(D)** Summary data of ACh induced relaxation. PTX group; *n* = 11 out of eight mice. 0.9% NaCl group; *n* = 7 out of four mice. ^∗^*P* < 0.05 using unpaired *t*-test; n.s., not significant.

**FIGURE 6:**
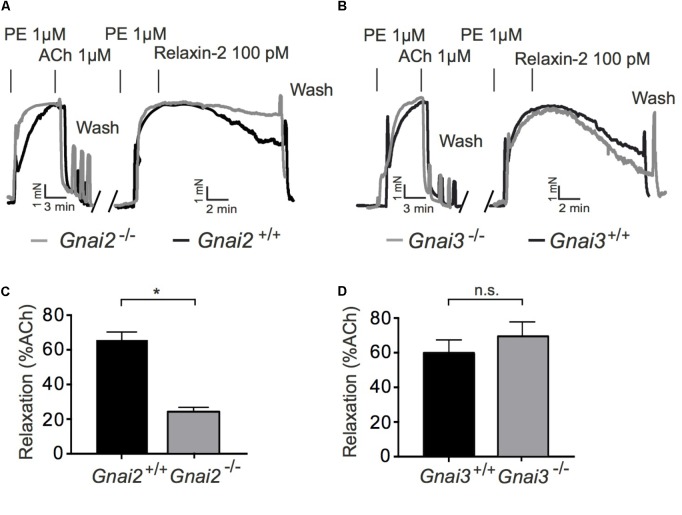
Vasorelaxant effects of relaxin-2 in Gα_i2_ deficient (*Gnai2*^-/-^), Gα_i3_ deficient (*Gnai3*^-/-^), and control arteries. **(A,B)** Original representative recordings. **(C)** Summary of data. Gα_i2_ deficient arteries; *n* = 9 out of five mice. Control; *n* = 9 out of four mice. **(D)** Summary of data. Gα_i3_ deficient arteries; *n* = 6 out of five mice. Control arteries; *n* = 7 out of four mice. ^∗^*P* < 0.05 using unpaired *t*-test.; n.s., not significant.

To determine a possible role of G_i_ proteins in relaxin-2-dependent relaxation, we treated mice with pertussis toxin (PTX), which is used as pan-Gα_i_-inhibitor *in vivo* (Devanathan et al., 2015). Control mice were treated with 0.9% NaCl only. **Figure 5A** shows that G_q_-dependent relaxations by ACh were preserved in mesenteric arteries treated with PTX. However, relaxation in response to relaxin-2 were abolished in mesenteric arteries from mice pre-treated with PTX (**Figures 5A,C**) compared to control arteries (**Figures 5B,C**). In contrast, relaxations in response to ACh were not changed by PTX treatment (**Figure 5D**). The G_i_-family comprises three closely related Gα members, Gα_i1-3_, with Gα_i2_ and Gα_i3_ abundantly expressed in the cardiovascular system (Hippe et al., 2015). We therefore used Gα_i2_-deficient (*Gnai2*^-/-^) and Gα_i3_-deficient (*Gnai3*^-/-^) mice to determine which Gα_i_ isoforms are involved in relaxin-2 mediated relaxations. **Figure 6** shows that relaxin-2 induced relaxations were reduced in Gα_i2_-deficient arteries (**Figures 6A,C**) but not in Gα_i3_-deficient arteries (**Figures 6B,D**). These data indicate that Gα_i2_ plays an important role in RXFP1 mediated relaxations.

### Involvement of PI3Kβ and PI3Kγ in Relaxin-2-Dependent Relaxations

**FIGURE 7:**
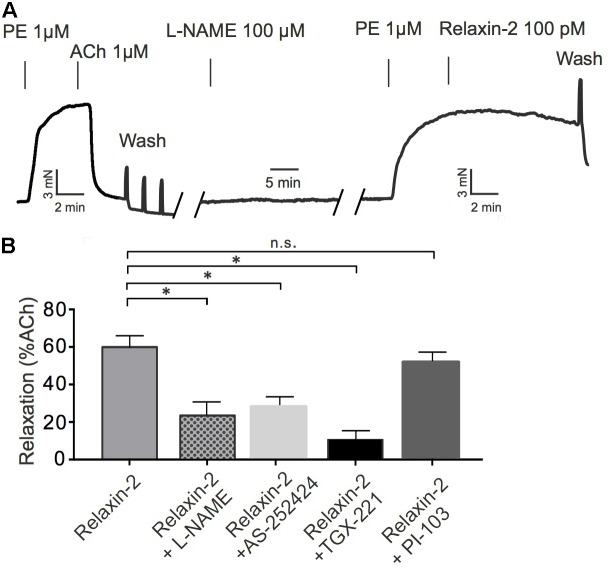
Effects of PI3K inhibitors on relaxin-2 induced relaxation. **(A)** Original representative recording. **(B)** Summary of data. Relaxin-2-induced relaxation in the presence of 100 μM L-NAME (*n* = 6 out of four mice), 100 nM AS-252424 (*n* = 7 out of four mice), 100 nM TGX-221 (*n* = 7 out of four mice), or 100 nM PI-103 (*n* = 6 out of four mice). ^∗^*P* < 0.05 using one-way ANOVA followed by Bonferroni multiple comparisons test; n.s., not significant.

PI3K signaling has been proposed to mediate slow and sustained activation of eNOS and subsequent NO release in cell culture experiments (Dimmeler et al., 1999). We tested the contribution of different PI3K isoforms in eNOS/NO-dependent RXFP1 receptor-mediated relaxations in comparison to ACh-induced relaxations. Vessels were pre-contracted with PE, incubated with L-NAME or various PI3K inhibitors and subsequently exposed to relaxin-2 (**Figure 7**). **Figures 7A,B** shows that relaxin-2-induced relaxations were inhibited by L-NAME. The PI3Kβ inhibitor TGX-221 (**Figure 7B**) and the PI3Kγ inhibitor AS-252424 (**Figure 7B**) had similar inhibitory effects on stimulation by relaxin-2. However, relaxin-2-induced relaxations were not affected by the PI3Kα inhibitor PI-103 (**Figure 7B**). Together, the data suggest that PI3K isoforms β and γ are essential intermediate signaling components in eNOS/NO-dependent relaxation by relaxin-2, which are controlled by relaxin-2 that act on RXFP1 receptors coupled to G_i2_ proteins.

### RXFP1 Activation by Relaxins Is Unlikely Involved in Arterial Tone Regulation by Perivascular Adipose Tissue

**FIGURE 8:**
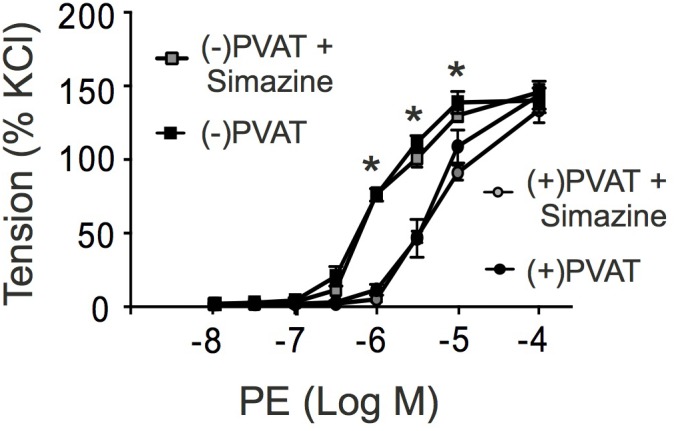
Effects of simazine on phenylephrine (PE)-dependent contractions in the presence (+) or absence (-) of perivascular adipose tissue (PVAT). Summary data for PE induced contractions in (+) PVAT (∙, *n* = 8 out of four mice) or (-) PVAT (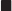, *n* = 8 out of four mice) rings. Incubation of the rings with simazine (100 nM, 30 min) had no effects on contractions caused by PE in (+) PVAT (∙, *n* = 8 out of four mice) and (-) PVAT (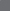, *n* = 9 out of 4 mice) arterial rings (*P* > 0.05 each). ^∗^*P* < 0.05 for (-) PVAT vs. (+) PVAT or (-) PVAT + Simazine vs. (+) PVAT + Simazine; repeated-measures two-way ANOVA, followed by Bonferroni *post hoc* test.

Perivascular adipose tissue (PVAT) plays a functional role in regulating the contractile state of arteries by production of numerous vasodilatory substances (Gollasch, 2017). Since relaxin(s) are expressed in adipose tissue (Hausman et al., 2006), these polypeptide hormones might represent an adipose-derived relaxing factor released by PVAT to contribute to anti-contractile effects on arterial tone. Therefore, we studied vascular contractions in response to PE in arterial ring in the presence and absence of PVAT. **Figure 8** shows that in the absence of PVAT phenylephrine was more potent in causing effective contractions of arterial rings than in the presence of PVAT. This anti-contractile effect of PVAT was not influenced by the incubation of the arteries with the RXFP1-receptor antagonist simazine (100 nM) (**Figure 8**). We also performed control experiments on whether simazine or the other drugs used in this study affect contractions caused by PE. The results in **Supplementary Figure S1** show that contractions caused by PE in the absence (first PE application) and presence of the respective drugs (second PE application) were not different. The data demonstrate that neither simazine nor the other drugs used in this study affected PE contractions.

## Discussion

Our study shows that relaxins are extremely potent (in the low picomolar range) endothelium-dependent and L-NAME-sensitive vasodilators in mouse mesenteric arteries. Similar vasodilatory potencies of relaxins have been observed renal arteries of rats (Novak et al., 2002) and human small gluteal and subcutaneous arteries (Fisher et al., 2002). Among the three human relaxins studied, we identified relaxin-2 as the most effective vasodilator, which produces eNOS/NO-dependent relaxation most likely due to activation of RXFP1 coupled to a G_i2_-PI3kβ/PI3kγ pathway. Although, we probed human relaxins in a non-human vascular preparation, that is, in isolated arteries from mice, this is the first study suggesting that RXFP1 coupled to a G_i2_-PI3kβ/PI3kγ pathway is capable of producing vascular relaxation. Furthermore, our data indicate that this pathway does not contribute to PVAT control of arterial tone.

### Relaxin Family of Peptides

Relaxin-encoding genes are present in all mammals and responsible for the production of the relaxin peptides that have been initially found in circulating blood during pregnancy. However, more recent studies have observed that relaxins are produced in many tissues in mammals as paracrine or autocrine factors to exert a number of different physiological roles in the vasculature, which may exhibit protective effects in cardiovascular disease (Samuel et al., 2006). The injection of recombinant human relaxins to normotensive rats for 1 to 6 h induced a systemic vasodilatory response (Debrah et al., 2005). This finding suggests that certain vascular beds, for example, in the kidney or mesentery, are able to respond by a vasodilatory response caused by relaxins. Relaxin-2 has been identified as the most important member of the relaxin family and major circulating form of relaxin peptides in humans (Grossman and Frishman, 2010). Relaxin-1 is also believed to exist as circulatory peptide in the circulation (Bathgate et al., 2013), but the function of the relaxin-1 in humans and higher primates is mostly unclear. Relaxin-3 is the most recently identified member of the relaxin family and is primarily expressed in the brain of mammals (Heidari et al., 2018). In our study, we first tested the vasoactive function of all three relaxins in mouse mesenteric arteries. We found that all three relaxins can produce relaxations, with relaxin-2 being the most effective member of the relaxin family. In contrast to ACh, which produces a rapid relaxation, relaxin-1, relaxin-2, and relaxin-3 induced slow and delayed relaxations, indicating different underlying signaling mechanisms between ACh and relaxins to cause endothelium-dependent relaxations.

### Relaxin-2 Exerts Relaxation by Activating RXFP1 in Endothelium

**FIGURE 9:**
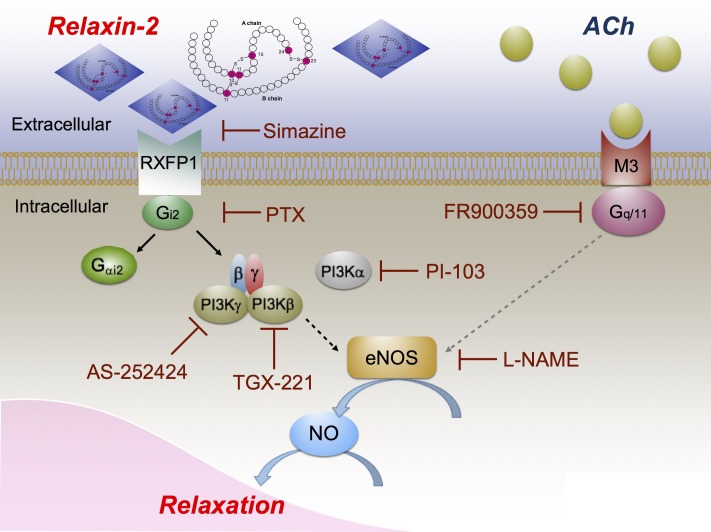
Proposed vasodilatory pathways caused by relaxin-2. Relaxin-2 activates RXFP1 (blocked by simazine), which leads to G_i2_ activation and dissociation of Gα_i2_ and βγ subunits (blocked by PTX). Gβγ subunits in turn activate PI3Kβ (blocked by TGX-221) and PI3Kγ (blocked by AS-252424) to initiate eNOS activation and NO release (blocked by L-NAME) to cause relaxation. PI3Kα (blocked by PI-103) seems not to be involved in this pathway. On the other hand, ACh binds to muscarinic M3 receptors coupled to G_q/11_ (blocked by FR900359) to produce eNOS/NO dependent arterial relaxation.

Studies on isolated vessels have shown that the endothelium is necessary for vasodilation by relaxin in renal and human subcutaneous arteries (McGuane et al., 2011). In our study on mouse mesenteric arteries, we also found that removal of the endothelium or treatment with L-NAME inhibited relaxation by relaxin-2. Our data support previous findings indicating that relaxins cause vasodilation primarily by an eNOS/NO-dependent mechanism (McGuane et al., 2011; Ng et al., 2015; Leo et al., 2016). Relaxin produces its major effects *via* specific G-protein-coupled receptors (GPCRs), that is, RXFP 1-4 (Hsu et al., 2002; Bathgate et al., 2006). Among them, RXFP1 was the first to be identified and remains in focus of interest because of its crucial role in the cardiovascular system (Bathgate et al., 2013). RXFP2 receptors are mainly activated by insulin-like (INSL) 3 in the gubernaculum to facilitate testicular descent into the scrotum, and RXFP3 and RXFP4 receptors are activated by relaxin-3 and INSL5, respectively (van der Westhuizen et al., 2008). RXFP1 has the highest binding potency for relaxin-2 within the several members of the relaxin peptide family (Samuel et al., 2006; Nistri et al., 2007). RXFP1 is predominantly expressed in endothelial cells of mesenteric arteries and veins, but also expressed in the aorta and vena cava (Novak et al., 2006; Jelinic et al., 2014). In our experiments, the RXFP1 antagonist simazine (Park et al., 2016) largely inhibited the endothelium-dependent relaxation induced by relaxin-2, which supports the idea that relaxin-2 produces relaxation primarily through binding to and activation of RXFP1 in the endothelium (**Figure 9**). The remaining relaxation of about 25% in simazine-treated vessels may represent spontaneous relaxation or caused by RXFP activation distinct from RXFP1 in the vasculature or both of them.

### Relaxin-2 Induces eNOS/NO-Dependent Vasodilation Through a G_i2_-PI3Kβ/PI3Kγ Pathway

We aimed to identify the G proteins coupled with RXFP1 to cause eNOS/NO-dependent vasodilation by relaxin-2. Heterotrimeric G proteins, which mediate signals from cell surface receptors to cellular effectors, are composed of α, β, and γ subunits, of which Gα defines the class of G proteins (Simon et al., 1991). The α subunits that define the basic properties of a heterotrimeric G protein can be divided into four families, namely G_s_, G_q_/G_11_, G_i_/G_o_, and G_12_/_13_ (Kaziro et al., 1988; Wettschureck and Offermanns, 2005). ACh produces relaxation mainly *via* the G_q_/G_11_-coupled M3 receptor subtype (**Figure 9**) (Jaiswal et al., 1991; Kruse et al., 2012). In our study, we found that ACh-induced relaxations were abolished by FR900359, a selective mammalian G_q_/G_11_ signaling inhibitor (Schrage et al., 2015), while the relaxin-2-induced relaxations were not blocked by this drug. We conclude that vasodilatory RXFP1-coupled G proteins are distinct from G_q_/G_11_ (**Figure 9**). Our data using the pan G_i_/G_o_ inhibitor PTX (Simon et al., 1991) indicate that G_i_/G_o_ proteins could play a major role in relaxin-2 mediated vasodilation. G_0_ is particularly abundant in the neuronal and the neuroendocrine system and the G_i_-family includes three closely related Gα members, Gα_i1-3_, which display overlapping expression patterns with Gα_i2_ and Gα_i3,_ abundantly expressed in the cardiovascular system (Hippe et al., 2015). According to previous studies on cultured cells, RXFP1 has been suggested to couple to Gα_i3_ to release Gβγ dimers to activate the PI3K pathway *via* Akt phosphorylation and subsequently initiate NOS (Halls et al., 2006; McGuane et al., 2011). Our experiments using Gα_i2_-deficient (*Gnai2*^-/-^) and Gα_i3_-deficient (*Gnai3*^-/-^) mice failed to implicate an important role of Gα_i3_, but revealed a key role of Gα_i2_ in RXFP1-eNOS/NO relaxation. We found that relaxin-2 induced relaxation was impaired in arteries from *Gnai2*^-/-^, but not from *Gnai3*^-/-^ mice. Although structural similarity between the three G_i_ subforms suggests that they may exhibit overlapping functions, Gα_i2_/Gα_i3_-double-deficient mice cannot be used for myography experiments because they die *in utero* at early embryonic stages. Nevertheless, present data obtained in mice lacking Gα_i2_ or Gα_i3_ indicate distinct biological key roles of these two Gα_i_-isoforms (Köhler et al., 2014). Thus, we believe that relaxin-2 relaxes mouse mesenteric arteries primarily *via* RXFP1 activation and coupling to G_i2_ but not G_i3_ (**Figure 9**). Moreover, since the pan G_i_/G_o_ inhibitor PTX shows a stronger inhibitory effect than the absence of Gα_i2_, we should also consider that relaxin-2 could partly act through other G_i_ proteins.

Following the release of Gβγ from Gα_i_, it was recently suggested that the class I PI3K represents a target for G_i_βγ signaling by relaxins (McGuane et al., 2011). Accordingly, we found that eNOS activation by relaxin was inhibited by the pan PI3K inhibitors Wortmannin or LY294002 in cultured endothelial cells (Dessauer and Nguyen, 2005). Based on the association with non-catalytic binding proteins, catalytic subunits of class I PI3Ks are subdivided into class IA-isoforms (p110α, -β, and -δ) or class IB p110γ (Hennessy et al., 2005). In this study, we found that relaxin-2-RXFP1 relaxation was inhibited by the PI3Kγ and PI3Kβ inhibitors AS-252424 and TGX-221, respectively. The PI3Kα inhibitor PI-103 had no effects. These data indicate that class I PI3Kγ and PI3Kβ represents likely a target for G_i2_ signaling by relaxins to cause eNOS/NO dependent relaxation. We are not aware of selective PI3kδ inhibitors to determine a possible additional role of PI3Kδ in RXFP1-mediated relaxation. Nevertheless, our data indicate that class I PI3K activation by a target for RXFP/Gi-βγ signaling to cause eNOS activation is not only a cell culture phenomenon (Dimmeler et al., 1999; Dessauer and Nguyen, 2005), but is important for relaxins to produce vascular relaxation (**Figure 9**). Of note, the relaxin-2-induced relaxations were not fully abolished by TGX-221 or AS-252424. This may indicate that both PI3K isoforms or another PI3K isoform are involved in this vasoregulatory pathway. Also, in addition to the slow activation process of the eNOS/NO by relaxins *via* G_i_-PI3K (McGuane et al., 2011), there is also an ultra-slow mechanism of eNOS/NO stimulation *via* κ upon exposure of cultured endothelial cells to relaxin (Dschietzig et al., 2003). Although this putative mechanism cannot be examined by the methodological approach used in our study, such non-RXFP-mediated effects may contribute to the effects of relaxins in the vasculature *in vivo*.

Microarray studies showed expression of relaxin in pig adipose tissue (Hausman et al., 2006). According to our previous studies, PVAT inhibits vessel contraction and produces endothelium-independent relaxation by releasing adipocyte-derived relaxing factor (ADRF) (Löhn et al., 2002; Dubrovska et al., 2004; Verlohren et al., 2004; Tsvetkov et al., 2016b). PVAT dysfunction is characterized by disturbed secretion of various adipokines, which, together with endothelial dysfunction, contribute to hypertension and cardiovascular risk (Lian and Gollasch, 2016). With our interest, we aimed to unravel whether relaxin(s) acting through RXFP1 receptors may represent an ADRF. Our data showed that the RXFP1 antagonist simazine does not influence the anti-contractile effects of PVAT, which indicates that it is unlikely that relaxin(s) acting though RXFP1 receptors contributes to PVAT control of arterial tone, at least in mouse mesenteric arteries.

### Limitations

There are a number of limitations in the present study. First, we studied human relaxins rather than mouse relaxins to identify G_i_ protein-dependent vasodilatory pathways in the murine vasculature. Utilizing the murine vasculature enabled us to use advance of transgenic mouse models. Since serelaxin (RLX030) represents the recombinant form of human relaxin-2, which shows quite reliable therapies in cardiovascular diseases (Papadopoulos et al., 2013; Parikh et al., 2013; Teerlink et al., 2013), we are aimed to identify the function and mechanisms of RXFP1signaling pathways utilized by human relaxins in the vasculature. Of note, human relaxin-2 (H2 relaxin) is the counterpart of mouse relaxin-1 (M1 relaxin) within the structurally related insulin/relaxin superfamily, and mouse RXFP1 shows 89% identity to human RXFP1 (Sherwood, 2004; Kong et al., 2010). Binding of relaxins to RXFP1 is mediated *via* high-affinity binding to extracellular domain of RXFP1 and an additional binding site in the transmembrane (TM) exoloops (Sherwood, 2004). A recent study has shown that specific residues in the center of the H2 relaxin A-chain are necessary for ligand activity at RXFP1 (Park et al., 2008). Importantly, modeling of the ligand–receptor interaction for different RXFP receptors suggests that once the B-chains of the ligands are bound to the primary binding site in a large ectodomain with 10 leucine-rich repeats (LRRs) that the A-chain is presented in a favorable orientation for interaction with the TM exoloops (Hartley et al., 2009). Hence, it is likely that human and murine relaxins utilize similar common mechanisms to activate RXFP1 receptors, although there might be species-dependent differences in the mode of interaction between the individual relaxins, extracellular RXFP1 domains and the TM exoloops of the individual receptors. Second, we used female instead of male *Gnai2*^-/-^ and *Gnai3*^-/-^ mice and respective littermate controls. Because of the estrous cycle in females, there could be gender-related differences of the vascular reactivity and endothelial function (Sader and Celermajer, 2002), which should be considered. Nevertheless, our data clearly demonstrate a genotype-dependent inhibition of relaxin-2 relaxations, that is, lack of relaxin-2 relaxation in arteries from *Gnai2*^-/-^, but not from *Gnai3*^-/-^ mice. Lastly, considering that inhibition of endothelium may have influence on PE pre-contraction levels, for example, by mitogen-activated protein kinase kinase/extracellular signal-regulated kinase-dependent mechanisms (Molnar et al., 2008), we compared relaxin relaxations with ACh relaxations also in KCl-precontracted vessels. Our data show relaxin-2 relaxations involve an RXFP1-G_i_ protein pathway in both conditions. The G_q_ inhibitor FR900359 did not inhibit relaxin-2 relaxations and PE contractions were not affected the drugs used in our study (**Supplementary Figure S1**). Nevertheless, removal of the endothelium may stabilize the level of pre-contraction to play an additional role in eliminating endothelium-dependent RXFP1 relaxations.

## Conclusion

In summary, we provide evidence that all three relaxins, that is, relaxin-1, relaxin-2, and relaxin-3, are potent vasodilators in mesenteric arteries of mice. Among them, relaxin-2 is the strongest vasodilator, which produces relaxation *via* activation of endothelial RXFP1 coupled to a G_i2_-PI3Kγ/β-eNOS/NO pathway. Based on the fact that long- and intermediate-distance conduction of vasodilation is common in the circulation, localized releases of relaxins within a tissue might be able to produce remote vasodilations in regions of reduced blood flow distribution. As a potent vasodilatory Gα_i2_-coupled receptor, targeting RXFP1 may represent a promising avenue to study G_i_-coupled receptor based drugs in cardiovascular disease that may allow clarifying specific roles for Gα_i2_ and Gα_i3_ in response to GPCR activation directly in the vasculature.

## Author Contributions

XL performed the wire myography experiments. XL and MG drafted the article. All authors planned and designed the experimental studies and contributed to its completion.

## Conflict of Interest Statement

The authors declare that the research was conducted in the absence of any commercial or financial relationships that could be construed as a potential conflict of interest.

## Supplementary Material

FIGURE S1 Contractions induced by phenylephrine (PE). Summary data of contractions induced by PE in the absence (first application) and presence (second) application of vehicle (control), simazine, L-NAME, AS252424, TGX-221, or PI-103. For concentrations and number of rings, see other figure legends. w/o endothelium; effects of vehicle in the absence of endothelium. In all other experiments, the endothelium was intact. n.s., not significant.
