# Supplementary figures and images for: RXFP1 Receptor Activation by Relaxin-2 Induces Vascular Relaxation in Mice via a Gαi2-Protein/PI3Kß/γ/Nitric Oxide-Coupled Pathway
Source: Front Physiol. 2018 Sep 4;9:1234. doi: 10.3389/fphys.2018.01234 (PMC6131674; doi:10.3389/fphys.2018.01234)

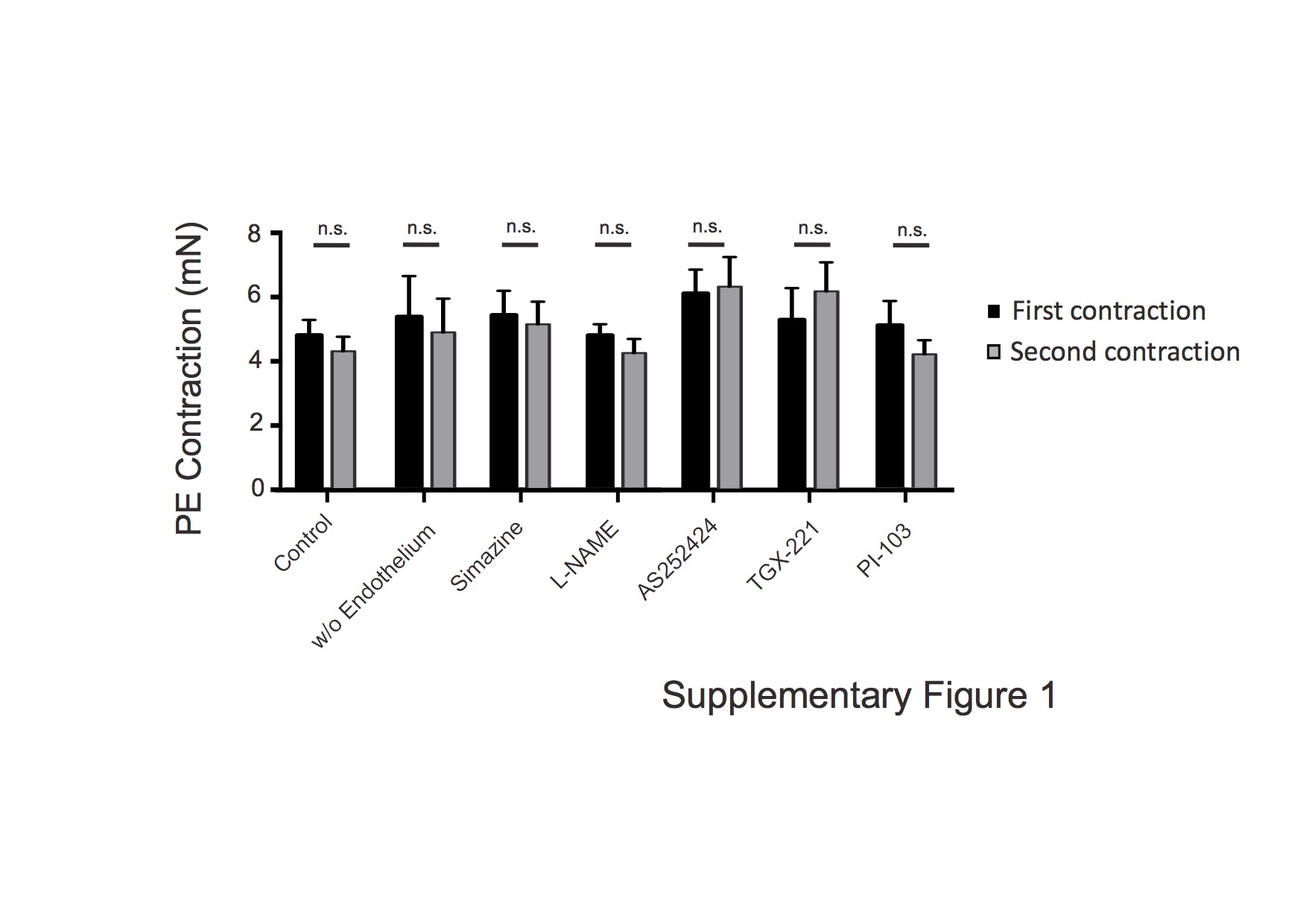

Supplement: FIGURE S1 — Contractions induced by phenylephrine (PE). Summary data of contractions induced by PE in the absence (first application) and presence (second) application of vehicle (control), simazine, L-NAME, AS252424, TGX-221, or PI-103. For concentrations and number of rings, see other figure legends. w/o endothelium; effects of vehicle in the absence of endothelium. In all other experiments, the endothelium was intact. n.s., not significant. [file Image_1.JPEG]
